# Thermal and Non-Thermal Treatments to Preserve and Encourage Bioactive Compounds in Fruit- and Vegetable-Based Products

**DOI:** 10.3390/foods11213400

**Published:** 2022-10-27

**Authors:** Marina Cano-Lamadrid, Francisco Artés-Hernández

**Affiliations:** Postharvest and Refrigeration Group, Department of Agronomical Engineering, Institute of Plant Biotechnology, Universidad Politécnica de Cartagena, 30203 Cartagena, Murcia, Spain

Fruit- and vegetable-based products (F&Vs) have been conventionally processed using thermal techniques such as pasteurization, scalding, or/and drying, ensuring microbial safety and/or enzyme deactivation. Although thermal treatments are the most cost-effective tools, they could also reduce bioactive compounds, nutrients, and even sensory attributes. Nowadays, non-thermal food-processing technologies such as UV light and high-pressure processing have been proposed to develop food products with extended shelf life and preserved/encouraged bioactive compounds, while preserving the sensory properties. To contribute to the knowledge of novel thermal and non-thermal techniques in the production of F&Vs and their derivatives, we present this Special Issue, “Thermal and Non-Thermal Treatments to Preserve and Encourage Bioactive Compounds in Fruit- and Vegetable-Based Products”. Among the 13 published papers, three main research topics have been covered: (i) thermal and non-thermal treatments on whole fruits; (ii) thermal and non-thermal treatments on fruit and vegetable byproducts; (iii) thermal and non-thermal treatments on fruit-based products (beverages and purées).

There are four papers within the first topic, dealing with the effect of thermal and non-thermal treatments on whole fruits [1,2,3,4]. The first study, by Bedrníček et al. [1], investigated the influence of several thermally treated fresh garlic varieties (treated during ageing process—15 days with a temperature gradient ranging from 30 to 82 °C) on the physicochemical parameters of black garlic. During this thermal process, the health-promoting properties of fresh garlic are usually enhanced, but very little information has previously been reported about how the physicochemical properties of fresh garlic varieties affect those same properties of black garlic. The authors concluded that the antioxidant activity, the total polyphenol content, and the total soluble solids increased during ageing, while the pH level, moisture content, and lightness decreased in all the garlic varieties. Texture acceptance was significantly affected among the fresh and black garlic varieties, while color, odor, taste, and the acceptance of the intensity of the garlic aroma were not affected. It was highlighted that the fresh garlic parameters did not correlate well with the properties of black garlic; therefore, they cannot be used for its quality prediction. The second study, a review by Darré et al. [2], showed that ultraviolet radiation has been found to be a germicide and a potentially damaging agent; however, beneficial properties have also been reported in harvested commodities. UV treatments on whole fruit were shown to induce phytochemicals accumulation, including ascorbic acid, carotenoids, glucosinolates, and, more frequently, phenolic compounds. Scientific evidence related to the effect of ultraviolet radiation on whole fruit and vegetables through several factors (product type, maturity, cultivar, UV spectral region, dose, intensity, and radiation exposure) was collected. Additionally, the authors posited a need to fix the optimal operational conditions to maximize UV radiation efficacy—reducing treatment times and ensuring even radiation exposure—especially under realistic processing conditions. The third study in this topic, performed by Modesti et al. [3], aimed to summarize knowledge about the possibility of improving bioactive compounds by using ozone; then, they applied these findings to the health-related properties of grapes and wine. The greatest focus was given to the pre- and post-harvest treatment of table and wine grapes, in addition to the explanation of the mechanisms involved in the ozone-related response and the main secondary metabolites biosynthetic pathways. The authors highlight that the effect of ozone treatment on health-related properties and secondary metabolites accumulation depends on many factors (cultivar, ozone type, doses, and application method). Scientific evidence reports an increase in bioactive compounds with antioxidant activity, improving berry and wine functional and sensory quality. The fourth study, a review by Jacobo-Velázquez et al. [4], identified technologies that emulate a wound response (increase the content of bioactive compounds and increase detrimental reactions in fruits and vegetables) in whole F&Vs while retaining their quality attributes. The application of non-thermal technologies, such as high hydrostatic pressure, ultrasound, and pulsed electric fields, are presented in this review as tools for increasing the content of health-promoting compounds in whole F&Vs by inducing a wound-like response. Additionally, the industrial implementation and economic feasibility of these approaches were discussed.

There are three manuscripts in this Special Issue addressing the second topic—different thermal and non-thermal treatments on F&V byproducts [5,6]. The first study of this topic, by Michalska-Ciechanowska et al. [5], aimed to produce chokeberry pomace extract powders and conduct experimental and chemometric assessment of the effect of different carriers (10% of maltodextrin, inulin, and trehalose, and their combinations) and drying techniques (freeze-drying and vacuum-drying at 60 °C or 90 °C) on the polyphenols, anthocyanins, flavonols, phenolic acids, and hydroxymethyl-L-furfural of chokeberry pomace powders. Drying techniques had a stronger effect on the bioactive compound profiles than the type of carrier. Hydroxymethyl-L-furfural formation was enhanced by inulin addition during high-temperature treatment. They concluded that the addition of maltodextrin and trehalose mixture for freeze-drying and vacuum-drying at 90 °C caused the highest retention of polyphenolics and the lowest formation of hydroxymethyl-L-furfural. The second study, a review by Cano-Lamadrid et al. [6], provided comprehensive information about non-thermal technologies applied in F&V byproducts to enhance their phytochemicals and to obtain pectin. The potential use of bioactive compounds for food supplementation has also been collected as sustainable strategy to increase functional properties. Applications of ultrasound, light stress, enzyme-assisted treatment, fermentation, electro-technologies, and high pressure, among others, were included in this review. Future investigations should focus on the optimization of “green” non-thermal and sustainable technologies on the F&V byproducts’ key compounds for the full utilization of raw materials in the food industry. The third study, by Cano-Lamadrid et al., evaluated the state of knowledge of and scientific evidence for the use of green extraction technologies (ultrasound-, microwave-, and enzymatic-assisted technologies) of bioactive compounds from pomegranate peel byproducts, and their potential application via the supplementation/fortification of vegetal matrixes to improve their quality, functional properties, and safety. Most studies are mainly focused on ultrasound extraction, which has been widely developed compared with microwave or enzymatic extractions; these should be studied in depth, accounting for their combinations. After extraction, pomegranate peel byproducts (in the form of powders, liquid extracts, and/or encapsulated, among others) have been incorporated into several food matrixes as a good tool to preserve “clean label” foods, improving their functional properties without altering their composition [7].

Five original papers have been published in this Special Issue which address the third topic—dealing with the effects of thermal and non-thermal treatments on fruit-based products (beverages and purées) [8,9,10,11,12,13]. In the first study, by Zhao et al. [8], the aim was to determine the effect of thermo-sonication combined with nisin and thermal pasteurization treatments on the quality attributes—including microbial and enzyme inactivation and the physicochemical, nutritional, functional, and sensory qualities—of orange juice. Apart from desirable bactericidal and enzyme inactivation effects and an improvement in the physicochemical properties and sensory quality, thermo-sonication combined with nisin increased the total polyphenols content (10.03%), total carotenoids (20.10%), and antioxidant capacity (51.10% and 10.58%, by ORAC and DPPH, respectively). In addition, total flavonoids and ascorbic acid were largely retained. In the second study, by López-Gámez et al. [9], the authors proposed the application of pulsed electric fields to carrots to obtain juices, purees, and oil-added purees with increased phenolic and carotenoid bio-accessibility. These carrot-based products were obtained by treating carrots with pulsed electric fields (five pulses of 3.5 kV·cm^−1^; 0.6 kJ·kg^−1^). Among the carrot-based products obtained, carrot juices exhibited the highest carotenoid and phenolic contents. However, caffeic and coumaric acid derivatives were highly sensitive to pulsed electric fields. The phenolic bio-accessibility reached 100% in purees obtained from the pulsed electric fields-treated carrots, whereas the further thermally treated oil-added purees exhibited the greatest carotenoid bio-accessibility (7.8%). The third study, by Artés-Hernández et al. [10], was focused on analyzing the effects of different green technologies (UV, high-pressure processing, pulsed electric fields, ultrasounds, and cold plasma) in beverage processing; they found a fortification effect (improvement of their extraction/bioaccessibility and/or different biosynthetic reactions that occurred during processing) on their health-promoting compounds. Fortification can be performed by several strategies, including physical elicitors (e.g., processing technologies), plant/algae extract supplementation, and fermentation with probiotics, among others. Thermal processing technologies are conventionally used to ensure the preservation of food safety with a long shelf life, but this frequently reduces nutritional and sensory quality. Nevertheless, green non-thermal technologies are being widely investigated to reduce costs and make possible more sustainable production processes without affecting the nutritional and sensory quality of beverages. The fourth study, by Salazar-Orbea et al. [11], aimed to discuss the positive and negative effects of thermal treatment and high-pressure processing on the stability of different polyphenol families in agro-food products obtained from strawberry and apple, two of the most-used fruits in food processing. The main findings showed that the phenolic content was affected by the processing, fruit type, polyphenol family, and storage conditions (time and temperature) of the final product. To increase shelf life, manufacturers are focusing on the optimal conditions for polyphenol stability (residual enzyme activity from HPP can affect polyphenols negatively) and product shelf life. The fifth study, by Tsikrika et al. [12], investigated the polyphenol oxidase (PPO) inactivation in five whole and peeled Irish potato cultivars using high-pressure processing (400 MPa and 600 MPa for 3 min). The highest PPO inactivation was observed after high-pressure processing at 600 MPa. No changes were observed on the total phenolic content, antioxidant activity, and glycemic indices of all the high-pressure-treated potatoes. Regarding individual phenolic acids, chlorogenic acid and ferulic acid were decreased in all studied varieties with a resulting increase in caffeic and quinic acid, and a variation in rutin and 4-coumaric acid levels depending on the cultivar and the sample type, respectively. Higher content of anthocyanins (pelargonidin-O-ferulorylrutinoside-O-hexoside and pelargonidin-O-rutinoside-O-hexoside) was observed in colored whole-potato varieties treated by high-pressure processing than those untreated. The sixth study, by Salar et al. [13], studied the effect of high hydrostatic pressure (450 and 600 MPa for 180 s) compared with thermal pasteurization (85 °C for 15 s) on the microbiological and phytochemical profile of citrus–maqui beverages. Additionally, the shelf life under refrigeration (4 °C) and room temperature (20 °C) was monitored for 90 days. Microbiological stability and physicochemical parameters were not different after processing or throughout the storage period. An increase in the reddish coloration was noticed during storage for those beverages treated by high hydrostatic pressure. Phenolic compounds showed little affect from the processing technique, being more stable when high hydrostatic pressure was used during processing. The authors concluded that high hydrostatic pressure technology could be an effective alternative to thermal treatments, achieving effective microbial inactivation and extending the shelf life of the juices by contributing to a better preservation of color and bioactive compounds. 

In summary, the 13 papers published in this Special Issue are a strong representation of the research activities addressing the topic thermal and non-thermal treatments for preserving and encouraging bioactive compounds in F&V commodities (whole, byproducts, and F&V-based products). Finally, most authors who have contributed to this issue concluded that further research in their topic is required if we are to optimize the conditions of “green” and sustainable technologies for maintaining the functionality and safety of fruit- and vegetable-based products.
